# Necroptosis in neurodegenerative diseases: a potential therapeutic target

**DOI:** 10.1038/cddis.2017.286

**Published:** 2017-06-29

**Authors:** Shuo Zhang, Mi-bo Tang, Hai-yang Luo, Chang-he Shi, Yu-ming Xu

**Affiliations:** 1 Department of Neurology, The First Affiliated Hospital of Zhengzhou University, Zhengzhou University, Zhengzhou 450000, China; 2 Institute of Clinical Medicine, The First Affiliated Hospital of Zhengzhou University, Zhengzhou University, Zhengzhou 450000, China

## Abstract

Neurodegenerative diseases are a group of chronic progressive disorders characterized by neuronal loss. Necroptosis, a recently discovered form of programmed cell death, is a cell death mechanism that has necrosis-like morphological characteristics. Necroptosis activation relies on the receptor-interacting protein (RIP) homology interaction motif (RHIM). A variety of RHIM-containing proteins transduce necroptotic signals from the cell trigger to the cell death mediators RIP3 and mixed lineage kinase domain-like protein (MLKL). RIP1 plays a particularly important and complex role in necroptotic cell death regulation ranging from cell death activation to inhibition, and these functions are often cell type and context dependent. Increasing evidence suggests that necroptosis plays an important role in the pathogenesis of neurodegenerative diseases. Moreover, small molecules such as necrostatin-1 are thought inhibit necroptotic signaling pathway. Understanding the precise mechanisms underlying necroptosis and its interactions with other cell death pathways in neurodegenerative diseases could provide significant therapeutic insights. The present review is aimed at summarizing the molecular mechanisms of necroptosis and highlighting the emerging evidence on necroptosis as a major driver of neuron cell death in neurodegenerative diseases.

## Facts

- Necroptosis is closely associated with the pathogenesis of different kinds of neurodegenerative disease.
- Necroptosis can be widely stimulated by tumor necrosis factor (TNF), other members of the TNF death ligand family (Fas and TNF-related apoptosis-inducing ligand (TRAIL)), interferons, Toll-like receptors (TLRs) signaling and viral infection via the DNA sensor DNAdependent activator of interferon regulatory factor (DAI). Various upstream signaling pathways share the common terminal mechanism executed by mixed lineage kinase domain-like proteins.
- Blocking necroptotic pathways with synthetic inhibitors or genetic manipulation mitigates neurodegenerative disease *in vitro* and *in vivo*, which suggests a promising therapeutic strategy for neurodegenerative disease.

## Open Questions

- How to monitor necroptosis in the diagnosis and prognosis of neurodegenerative disease?
- What are the relative contributions of necroptosis and other forms of programmed cell death to neurodegenerative disease?
- Would synthetic inhibitors be the most effective against necroptosis-associated neurodegenerative disease in clinical settings?

Historically, two forms of cell death have been recognized: necrosis and apoptosis. They play essential roles in development, homeostasis, and pathogenesis.^1^ Traditionally, necrosis has been considered as an accidental death resulting from an over-whelming cytotoxic insult, and requires no specific molecular events. In contrast, apoptosis, which is characterized by apoptotic body formation, nuclear shrinkage and fragmentation, and membrane blebbing, had been researched as the main form of programmed cell death.^2^ However, increasing studies have described a genetically programmed and regulated form of necrosis, termed necroptosis.^3, 4^ Necroptosis can be triggered by the ligands of the death receptor family and extracellular and intracellular stimuli that induce their expression. The receptor-interacting kinase 3 (RIP3)^5, 6, 7^ and its substrate, the pseudokinase mixed lineage kinase domain-like protein (MLKL)^8, 9^ have been discovered to be core components of necroptotic signaling pathway. Morphologically, necroptosis resembles cellular necrosis, which is distinguished from apoptosis by the presence of clusters of dying cells, an early loss of plasma membrane integrity, cell and organelle swelling, granular cytoplasm, chromatin fragmentation, and cellular lysis.^10^ In contrast to apoptosis, the cellular contents of necroptotic cells passively enter the extracellular matrix through the disrupted cell membrane.^10^

**Figure 1:**
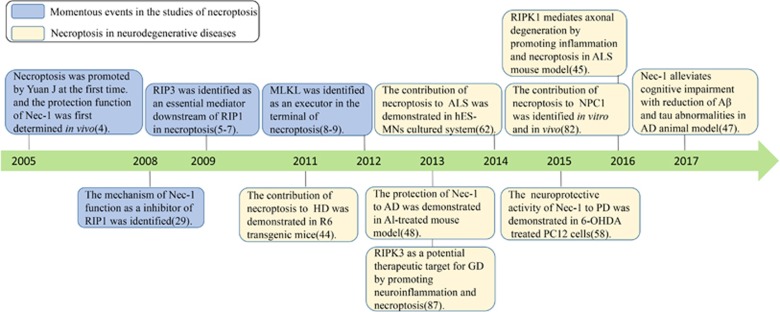
Timeline for momentous events in the studies of necroptosis and its role in neurodegenerative diseases. Blue boxes represent the major findings in the discovery of necroptosis signaling pathway; yellow boxes highlight the research breakthroughs concerning the role of necroptosis in neurodegenerative disease

Necroptosis is involved in many pathological processes, such as ischemia-reperfusion injury in the heart,^11^ brain,^4^ and in injury-induced inflammatory diseases.^12^ Necroptosis reportedly plays a critical role in the pathogenesis of several neurodegenerative diseases (Figure 1). Researchers have focused their attention on necroptosis due to its potential as a target for intervention in neurodegenerative diseases. In this review, we will discuss the molecular mechanisms of necroptosis and examine the growing evidence favoring a role for necroptosis in the development and progression of neurodegenerative diseases. We will also describe and comment on the effects of necroptosis in disease pathogenesis that may encourage research that will help in seeking therapies for neurodegenerative diseases.

## Activation of Necroptosis and Formation of Necrosome

**Figure 2:**
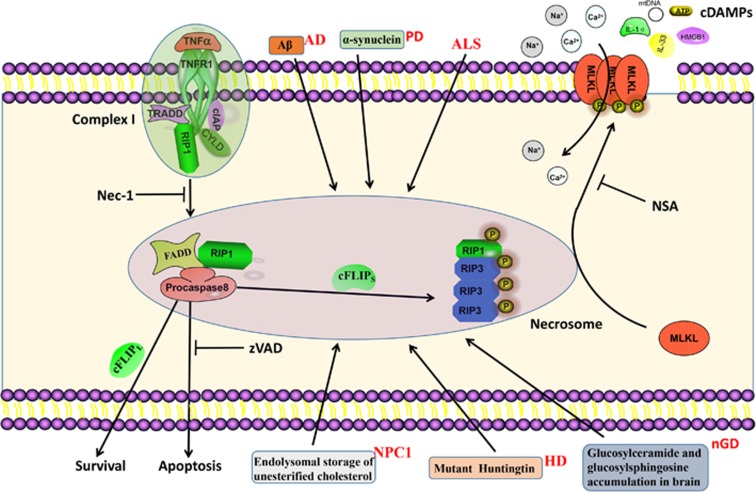
Necroptotic pathways. After TNF stimulation, TNFR1 recruits TRADD and RIP1 to complex I via their respective death domains. TRADD recruits cellular inhibitors of apoptosis (cIAPs), which ubiquitinate components of complex I. Deficient complex I activity, such as occurs with the deubiquitination of RIP1 by cylindromatosis (CYLD), can lead to the formation of the necrosome, which consist of FADD, procaspase-8, RIP1, RIP3, and MLKL. RIP1 interacts with FADD though death domain. Subsequently, procaspase-8, cFLIP_L_ and cFLIP_S_ are recruited to death receptor-bound FADD. Procaspase-8 homodimerization generate processed active caspase-8, which activates effector caspase cascade and induces apoptosis. Procaspase-8-cFLIP_L_ heterodimer does not process caspase-8 but cleaves RIP1, which leading to cellular survival. However, procaspase-8-cFLIP_S_ heterodimer fails to cleave RIP1, which allows the assembly of necrosome and the execution of necroptosis. In the necrosome, MLKL are phosphorylated and translocate to the plasma membrane, which leads to influx of Ca^2+^ or Na^+^ ions and direct pore formation with the release of cell damage-associated molecular patterns (cDAMPs) such as mitochondrial DNA (mtDNA), high-mobility group box 1 (HMGB1), interleukin (IL)-33, IL-1*α*, and ATP. zVAD, a pan-caspase inhibitors, inhibits caspases cascade and apoptosis. Nec-1 inhibits RIP1 kinase activity and necroptosis. NSA blocks necroptosis by preventing the membrane translocation of MLKL. Boxes around the cell model diagram represent the major pathogenesis of the neurodegenerative diseases in this review except ALS, of which the etiology remains unknown

Currently, one of the best studied form of necroptotic cell death is initiated by tumor necrosis factor (TNF), but necroptosis can also be induced by other members of the TNF death ligand family (Fas and TNF-related apoptosis-inducing ligand (TRAIL)), interferons (IFNs), Toll-like receptors (TLRs) signaling and viral infection via the DNA sensor DNAdependent activator of interferon regulatory factor (DAI).^3^ Here we take TNF as an instance to elaborate on the signal pathway of necroptosis. TNF activate TNF receptor 1 (TNFR1) through the pre-ligand assembly domain in the extracellular portion of TNFR1 and then triggers the trimerization of TNFR1.^13^ This process, then initiates the assembly of a transient molecular complex named complex I, which consists of TNFR1-associated death domain protein (TRADD), TRAF2, cellular inhibitor of apoptosis protein 1/2 (cIAP1/2) and RIP1^14,15^ (Figure 2). RIP1 was originally identified as a protein which interacted with TNFR1 signaling complex. Recent studies have revealed that in addition to being a regulator of necroptosis, RIP1 also participates in regulating inflammation and apotosis.^16, 17, 18^

In complex I, RIP1 is rapidly ubiquitinated by E3 ligases such as cIAP1.^3^ Ubiquitination of RIP1 plays an important role in regulating its kinase activity. Blocking RIP1 ubiquitination by antagonizing cIAP1/2 increases the sensitivity of cells to TNF-induced necroptosis.^19, 20, 21^ Cylindromatosis (CYLD), a K63-specific deubiquitinating enzyme,^22^ mediates the deubiquitination of RIP1 to facilitate the formation of complex IIb, which also termed necrosome,^23, 24^ which consists of RIP1, RIP3, and MLKL.^6, 7^ FADD and procaspase-8 can also be detected in necrosome^25^ (Figure 2). Recently, c-FLIP (caspase 8 and FADD-like apoptosis regulator), a catalytically inactive homolog of caspase-8, was reported to participate in the regulation of necroptosis.^26^ When a heterodimer is formed with c-FLIP long (c-FLIP_L_), caspase-8 maintains sufficient proteolytic activity to prevent the association of RIP1, RIP3 and FADD, thus inhibiting necroptosis.^19^ However, when caspase-8 is combined with c-FLIP short (c-FLIP_S_), it has no proteolytic activity, which allows the assembly of RIP1 and RIP3 and thus promotes necroptosis^19^ (Figure 2). In the absence of CYLD, necrosome formation is significantly inhibited in TNF-induced programmed necrosis.^3^ The activation of RIP1 leads to the recruitment of RIP3, a critical downstream mediator of necroptosis. RIP1 interacts with RIP3 through the RHIM motif to form an amyloid-like signaling complex in necroptotic cells.^27^ A critical consequence of RIP1 interaction with RIP3 is the phosphorylation of the latter.^5, 6, 7^ Enforced dimerization of RIP3 can also directly lead to its autophosphorylation.^28^ Necrostatin-1 (Nec-1),^29^ Nec-1s^30^ and other small molecules^31, 32^ were identified as small-molecule inhibitors of necroptotic signaling pathway, and they have been widely used to study the molecular mechanisms of necroptosis (Table 1).

## Execution of necroptosis

MLKL, which is detected in necrosome, is the most downstream effector of necroptosis identified so far.^9, 33^ MLKL contains an N-terminus coiled-coil domain region and a C-terminal kinase-like domain which binds the kinase domain of RIP3.^9^ The drug necrosulfonamide (NSA) was found to target MLKL and inhibit necroptosis.^9, 32^ NSA prevent the membrane translocation of MLKL, but has no effect on its phosphorylation or oligomerization.^9, 34^ The binding of RIP3 to MLKL is dependent on RIP3 kinase activity and the phosphorylation of RIP3.^9^ In addition, MLKL is phosphorylated by RIP3.^9^ Structural studies of MLKL allowed the identification of functionally important residues in MLKL. Mutations in ATP binding region of MLKL protein result in a constitutively active MLKL.^35^ A similar effect was observed for a phospho-mimic of one of the sites targeted by RIP3.^35^ These mutants can cause necroptosis in unstimulated cells, indicating the importance of MLKL modification in the necroptosis pathway downstream of RIP3 (ref. 35) MLKL deficient cells can undergo apoptosis but they are resistant to TNF-induced necroptotic cell death, as well as to LPS, oxLDL and partially cyclohexamide necroptosis, indicating that MLKL is a common downstream effector of different necroptotic death pathways.^33^ Recent studies reported that besides participate in necroptosis active MLKL also triggers the assembly of NLRP3 inflammasome in a cell-intrinsic manner, which is required for the activity of IL-1*β* released during necroptosis.^36, 37^

Recently, several researches have explored the role of MLKL in necroptosis.^8, 9^ MLKL oligomerizes through its N-terminal four-helix bundle, which triggers its translocation to the plasma membrane.^34^ Oligomerization of MLKL is induced by RIP3 mediated phosphorylation at the kinase-like domain of MLKL.^9^ The mechanisms of MLKL underlying necroptosis are not completely clear. One study reported that MLKL in the plasma membrane binds to the transient receptor potential melastatin-related 7 (TRMP7) ion channel, which leads to the influx of Ca2^+^ ions and induced cell death eventually^38^ (Figure 2). Another study stated that MLKL complex acts either by itself or with other proteins to increase the sodium influx by regulating the Na^+^ channels that triggers Na^+^ entrance, which increases osmotic pressure of cytoplasm, eventually leading to membrane rupture.^39^ Meanwhile, necroptosis causes severe inflammation through the release of cell damage-associated molecular patterns (cDAMPs),^40^ including mitochondrial DNA (mtDNA), high-mobility group box 1 (HMGB1), interleukin (IL)-33, IL-1*α*, and ATP^41^(Figure 2). In addition to the mechanism mentioned above, there are several studies showed that MLKL may be directly responsible for pore formation and membrane disruption.^34, 42^

## Necroptosis in Neurodegenerative Disease

Increasing evidence indicates that necroptosis may contribute to neuron death in several neurodegenerative disorders (Table 2). The signaling cascades leading to the organization of the necrosome can be inhibited by small molecules (Table 1). Treatment with Nec-1 was reported to be neuroprotective in cerebral ischemia^4^ and brain trauma.^43^ Furthermore, Nec-1 has been shown to be efficacious to target the necroptosis pathway in animal models of Huntington disease (HD)^44^ and Amyotrophic lateral sclerosis (ALS).^45^ In this review, we investigate the role of necroptosis in neurodegenerative disorders and evaluate the potential of inhibiting necroptotic signaling pathway as a therapy for neurodegenerative disorders.

## Alzheimer's Disease

Alzheimer's disease (AD), which is the most common neurodegenerative disease, is characterized by the accumulation of misfolded *β*-amyloid peptide (A*β*) plaques in the brain and neurofibrillary tangles composed of phosphorylated tau protein.^46^ However, the exact cause for AD is still not well understood. Recently, several studies have suggested that necroptosis may participate in AD.^47, 48^

Cells and animals treated with aluminum (Al) exhibite a significantly reductions in neuronal viability, neurofibrillary tangle formation, and AD symptoms, which might serve as a model of AD.^49, 50, 51, 52, 53^ Nec-1 might protect against Al-induced cell death by reducing the phosphorylation of RIP1. Moreover, Nec-1 improved the viability of Al-treated cell to a greater extent than zVAD-fmk and 3-methyladenine, which inhibit apoptosis and autophagy respectively.^48^ A study reported a similar conclusion: necroptosis is involved in Al-induced neuroblastoma cell death.^50^ Al-treated mice also showed a notable increase of neuron viability after they were treated with Nec-1, the behavior of animal were obviously better than controls, levels of RIP1 and AD-related proteins such as A*β* and Tau were significantly and dose-dependently decreased in the brains of mice model.^48^ These results suggest that necroptosis represents a potentially important pathway in AD pathogenesis. Nec-1 might slow the progression of the cognitive deficits associated with AD.

Consistently, Yang *et al.* examined amyloid precursor protein/presenilin-1 double-transgenic mice and reported that Nec-1 regulated multiple pathological culprits that are important in AD.^47^ Moreover, bimolecular binding between RIP kinase and A*β* was observed.^47^ Interaction between these two proteins in the extracellular and luminal regions might be involved in A*β*-related cell death pathology in AD and therefore might serve as a potential drug target for the disease.^47^ Further studies are needed to better understand how Nec-1 can selectively target and regulate A*β* and tau aggregation and investigate whether the therapeutic effects of Nec-1 translate into an intervention that might be potentially useful for AD.

## Parkinson’s Disease

Parkinson’s disease (PD) is a common neurodegenerative disorder with no known cure, estimated to affect 4 million people worldwide. It is characterized by the loss of dopaminergic neurons in striatum and substantia nigra and accumulation of modified *α*-synuclein in the degenerating neurons termed as Lewy bodies.^54^ However, the specific molecular events that occur during cell death in PD remains unclear. Recently, evidence has indicated that autophagy, which is downstream of necroptosis, is involved in dopaminergic cell death in PD.^55, 56, 57^

Wu *et al.* reported that Nec-1 could block necroptosis and give protection to dopaminergic neurons.^58^ They used 6-Hydroxydopamine (6-OHDA)-induced PC12 cells as a PD model^59^ to explore the role of necroptosis in PD by examining autophagic activation. Mitochondrial disability in PD cell model induced overactive autophagy, increased cathepsin B expression, and diminished Bcl-2 expression.^58^ Within a concentration of 5-30 *μ*M, Nec-1 increased the viability of the PC12 cells, stabilized the mitochondrial membrane potential, inhibited excessive autophagy, reduced the expression of cathepsin B and LC3-II, and increased Bcl-2 expression.^58^ However, when the concentration of Nec-1 was increased to 60 and 90 *μ*M, PC12 cells viability decreased, indicating dual effects of Nec-1, protection at lower concentrations *versus* toxicity at higher concentrations.^58^ These results were consistent with a point of discussion made by Smith and Yellon.^60^

These findings suggested that Nec-1 was neuroprotective against dopaminergic neuronal injury. The mitochondrial function-protective effects suggested that Nec-1 might have an antiapoptotic effect by stabilizing the mitochondrial membrane.^58^ The reversed effects on the levels of expression of LC3-II and cathepsin B in 6-OHDA-treated PC12 cells suggested that Nec-1 prevents autophagic cell death and downstream necroptotic signaling in the PC12 cells.^58^ However, based on these limited findings, it is still unknown whether Nec-1 has a protective effect in PD animal models. *In vivo* testing of Nec-1 will undoubtedly be the key to understanding its neuroprotective efficacy in PD.

## Amyotrophic Lateral Sclerosis

ALS is a neurodegenerative disorder characterized by the degeneration of motor neurons in the spinal cord, cortex and brain stem, leading to muscular atrophy and paralysis.^61^ The cell death mechanism in ALS has long been unclear. Recently, researchers have found that necroptosis is involved in neuron death in ALS.^45, 62^ In these studies, a humanized model was created by co-culturing human embryonic stem-cell-derived motor neurons (hES-MNs) with astrocytes collected from the motor cortex and spinal cord of ALS patients after death.^62^ The ALS astrocyte hES-MNs co-culture exhibited the ALS features of non-cell autonomous astrocyte-induced toxicity and was specific to motor neurons.^62^ In the co-cultures, silencing of neither SOD1 nor TDP-43 did not rescue motor neuron viability,^62^ providing evidence that neither misfolded SOD1 nor TDP-43 are necessary cytotoxic factors in sALS pathogenesis, which is contradict the results of other group.^63^

Researchers performed different cell death assays in the co-culture system. The sALS astrocyte hES-MNs co-culture showed significant DNA fragmentation, caspase-3 activation and decreased plasma membrane integrity, which showing evidence for both apoptosis and un-programmed/programmed necrosis.^62^ To further investigate this issue, inhibitors were used to block different cell death pathways. Unexpectedly, zVAD-fmk, a pan-caspase inhibitor, effectively eliminated caspase-3 activation, had no effect on the number of surviving motor neurons, indicating a caspase-independent cell death mechanism.^62^ These results conflict with a previous study that caspase inhibition prevents motor neuron cell death in mSOD1 mouse models,^64^ perhaps indicating that the cell death mechanisms in mSOD1 and sALS may be distinct. Further studies are needed to adequately address such issues. As described above, caspase-independent programmed cell death is strong evidence to suggest programmed necrosis and perhaps necroptosis. Treatment with Nec-1 further prevented motor neuron cell death to control levels.^62^ These results were confirmed by *RIP1* knockdown *in vitro*. Treatment with NSA resulted in similar levels of motor neuron viability as controls, which suggested that inhibiting MLKL prevented motor neurons from undergoing cell death.^62^ Thus, these results provide preliminary evidence that the necroptosis pathway may has a major role in driving motor neuron death in sALS. However, the evidence was generated *in vitro*.

Mutations in the optineurin (*OPTN*) gene have been implicated in both fALS and sALS pathogenesis.^65, 66, 67^ Ito *et al.* have shown that *OPTN^−/^^−^* mice have a decreased number of motor axons, abnormal myelination in the ventrolateral spinal cord, and a mild behavior phonetype.^45^ However, the total number and the bodies of neurons cell remained unaffected, researcheres considered that *OPTN* deficiency sensitizes cells to cell death in the spinal cord white matter of *OPTN^−/^^−^* mice.^45^ A subsequent study found that RIP1, RIP3 and MLKL were upregulated in the spinal cords of *OPTN^−/^^−^* mice and in SOD1 mice model,^45^ and increased levels of RIP3, along with activated RIP1 and MLKL were found in spinal cords of post-mortem ALS patients,^45^ which suggests that necroptosis may participate in the pathological process of ALS.^45^ Lineage-specific deletion of *OPTN* showed that it is oligodendrocytes and microglia that reproduced motor axon pathology in OPYN knockout mice.^45^ Researchers noted that *OPTN*^*–/–*^ oligodendrocytes were sensitized to die by TNF*α*-induced necroptosis but were protected by Nec-1s and in *OPTN*^*–/–*^*; RIP1*^*D138N/D138N*^ and *OPTN*^*–/–*^*;RIP3*^*–/–*^ double mutants.^18, 45, 68^ Thus, OPTN deficiency can promote necroptosis of oligodendrocytes. Moreover, the *OPTN*-knockout microglia exhibited a proinflammatory state and low expression levels of MLKL and increased levels of phosphorylated RIP1,^45^ which suggests that RIP1 activation in microglia may promotes inflammatory signaling not necroptosis.

Consistent with this hypothesis, an increased production of multiple proinflammatory cytokines, including interleukins IL-1a, IL-1b, IL-2, and IL-12; interferon-g (IFN-g); and TNF*α* in the spinal cords of *OPTN^−/^^−^* mice were observed.^45^ However, the expression of these proinflammatory cytokines were markedly reduced in the *OPTN^−/^^−^*;*RIP1*^*D138N/D138N*^ mice. The elevated TNF*α* in *OPTN^−/^^−^* microglia can be inhibited by Nec-1s.^45^ In this study, Nec-1s was used as the RIP1 inhibitor instead of Nec-1. Another study examined three Nec-1 analogs: Nec-1, Nec-1 inactive (Nec-1i), its inactive variant, and Nec-1s,^29, 69^ its more stable variant.^30^ Both Nec-1 and Nec-1i inhibited human indoleamine 2,3-dioxygenase (IDO),^70^ a potent immunomodulatory enzyme, but Nec-1s did not.^30^ Therefore, Nec-1s appears to be a more specific inhibitor of RIP1 and to lack the IDO-targeting effect. Next, Nec-1i was shown to be less effective compared with Nec-1 and Nec-1s in the inhibition of RIP1 kinase activity *in vitro* and *in vivo*.^30^ Importantly, low doses of Nec-1s did not exhibit cytotoxicity rather than Nec-1 or Nec-1i.^30^ Thus, it is suggests that the use of Nec-1s may provide a perfect alternative to the inhibition of RIP1. More studies are needed to determine whether *OPTN* deficiency results in inflammation. These results indicate that *OPTN* deficiency leads to motor axon defects by driving necroptosis in oligodendrocytes and/or a proinflammatory phenotype in microglia.^45^ Taken together, results above suggest a connection between RIP1-regulated necroptosis and inflammation. By regulating both inflammation and necroptosis, RIP1 may be a common mediator of axonal pathology in ALS, blocking RIP1 may be an effective intervention for the treatment of ALS.

## Huntington’s Disease

HD is characterized clinically by movement abnormalities, psychiatric symptoms, and cognitive deficits. Mutant Huntingtin (Htt) with expanded polyQ (glutamine) repeats causes the dysfunction and death of neurons, particularly striatal neurons.^71^ The mechanism underlying the striatal cell death remains elusive and no effective treatments are available for this fatal disease.

ST14A is an immortalized striatal cell line with medium spiny neurons (MSNs) characteristics^72^ and ST14A 8plx line stably expressing mutant Htt fragment was established as a cell model of HD.^73, 74^ Inhibition of death receptor signaling by zVAD-fmk, a pan-caspase inhibitor in apoptosis, leads to RIP1 kinase activation and cell death in ST14A and ST14A 8plx line cells, which can be almost completely rescued by Nec-1 (ref. 44) Unlike apoptosis, dying striatal cells showed atrophy and shrinkage of the cell body and had no caspase-3-specific cleavage. Nec-5, another RIP1 inhibitor,^29^ can not inhibit zVAD-fmk induced striatal cell death, indicates that there may be an alternative intrinsic RIP1 activation pathway in striatal cells.^44^

Investigator evaluated the Nec-1 in R6/2 transgenic mouse model of HD, which expresses exon 1 of mutant human htt gene.^75^ Given that Nec-1 can cross the blood-brain barrier easily but has a short half-life, about 1 h,^76^ Zhu *et al.* delivered Nec-1 intracerebroventricularly with Alzet osmotic pump to ensure continuous supply of the drug.^44^ Consequently, the expression of full-length RIP1 protein is increased in R6/2 mice compared with wild-type control.^44^ Nec-1 treatment helped maintain the motor functions and body weights in the R6/2 mice and significantly delayed disease onset. However, the survival benefit was modest.^44^ This discrepancy might be due to different mechanisms involved in early and late disease stages. Researches showed that apoptotic characteristics can be detected in late stage of R6/2 mouse and in grade 3 and grade 4 patients’ brain.^77^ The differentiation between early and late stages of the disease may due to different sensitivity of mutant striatal cells to excitotoxicity.^44^ According to Zuccato et al., the extensive and early involvement of activated astrocytes in HD pathogenesis might be induced by the necroptotic activation in early disease stage.^71^ In ST14A cells, Nec-1 treatment increased the cleavage of full-length RIP1, indicating the higher basal caspase-8 activity, which might have a side effect in the late apoptotic stage of the disease in mice.^44^ As RIP1 protein is also involved in caspase-8 activation in apoptosis, the interplay of apoptosis and necroptosis is even more complicated.^78^ Hence, concomitant treatment with both apoptosis and necroptosis inhibitors may have better therapy effect on HD. Finally, as Nec-1 helped ameliorates symptoms in R6/2 mouse, it can be considered as a potential treatment of HD patients.

## Niemann-Pick Disease

Niemann-Pick disease type C (NPC) is an autosomal recessive lysosomal lipid storage disorder with progressive neurodegeneration.^79^ NPC is classified as type C1 (NPC1) or type C2 (NPC2), which are caused by mutations in the NPC1 or NPC2 genes, respectively. Mutations in the NPC1 gene account for 95% of NPC patients.^80^ Degeneration of cerebellar Purkinje neurons is a prominent early feature in the disease progression, which leads to clinical symptoms of motor impairments.^81^ Very little is known about the cellular death mechanisms leading to neuronal loss in NPC1, and thus the potential efficacy of cell death inhibitors remains unexplored. A recent study reported that activation of the necroptotic pathway contributes to neuronal death in NPC1^82^ The expression level of RIP1 and RIP3 are raised with the formation of the necrosome in NPC1 fibroblasts.^82^ Consist with the results in NPC1-mutant mice and human patients brain tissue, thus strongly suggesting that necroptosis has a pathological role in NPC1.^82^ Of note, the formation of the necrosomal complex appeared to be more prominent in fibroblast lines from subjects with age adjusted neurological severity scores^83^ less than or equal to 1.5.^82^ Indicates that activation of the necroptotic pathway may be correlate with disease severity.

The mechanism by mutant NPC1 influences the disease are not fully understood. It is clear that necroptosis activation occurs during NPC1 progression, with an abundance of RIP1 and RIP3 found in NPC1 fibroblasts and post-mortem brain tissue of NPC patients compared to controls.^82^ Treatment of NPC1 fibroblasts from NPC1 patient with Nec-1 or suppression of either RIP1 or RIP3 expression can significant suppress cell death.^82^ Treatment of *Npc1^−/^^−^* mice with Nec-1 resulted in delayed cerebellar Purkinje cell loss, delayed progression of neurological manifestations and significantly prolonged lifespan.^82^ These results provide a strong link between necroptosis with the molecular mechanism that contributes to neuronal loss in NPC1.

## Gaucher’s Disease

Gaucher's disease (GD) is an inherited metabolic disorder caused by mutations in the gene encoding glucocerebrosidase and is the most common lysosomal storage disease.^84^ Impairment of glucocerebrosidase activity leads to accumulation of the sphingolipid glucosylceramide, which results in disease pathology via unknown mechanisms. The neuronopathic forms (types 2 and 3), which comprise 6% of patients with GD, are characterized by neuronal loss, microgliosis, and astrocytosis.^85^ Little is known about the molecular events leading to neuronal death.

Researchers detected profound levels of cell death in the cerebral cortex of *Gba*^*flox/flox*^; nestin-Cre mice, and in a conduritol-*β*-epoxide (CBE) induced mice model of GD.^86^ However, no signs of apoptosis were observed.^87^ Similarly, there was no elevation in caspase-9 or caspase-3/7 activity, and no cleavage of caspase-8.^87^ This suggests that neuronal cell death in nGD is caspase independent and nonapoptotic. Vitner et al. reported that levels of RIP1 and RIP3 were markedly elevated in the brains of symptomatic *Gba*^*flox/flox*^; nestin-Cre mice. Moreover, they detected elevated levels of c-FLIPs in the brains of symptomatic mice,^87^ suggesting the presence of a caspase-8-cFLIPs heterodimer, which would explain the lack of caspase-8 activity.^88^ Importantly, the expression of RIP1 was also elevated in brain of a patient who succumbed to type 2 GD compared to an age-matched control brain.^87^ These results indicate that necroptosis may play an important role in nGD brains. Moreover, RIP3 was mainly expressed in the nuclei of neurons from *Gba*^*flox/flox*^; nestin-Cre mice, in contrast to control mice where it was located in the cytoplasm, which implies a possible role for RIP3 in neuronal cell death.^87^

*RIP3*-deficient mice treated with CBE were used to explore the role of RIP kinases in GD pathology. *RIP1*-null mice die early after birth and are unsuitable for research.^89^ Glucocerebrosidase activity is inhibited in all cell types and organs upon CBE treatment,^90, 91^ in contrast to the *Gba*^*flox/flox*^; nestin-Cre mouse model in which glucocerebrosidase deficiency is restricted to cells of neuronal lineage. Levels of RIP1/RIP3, and c-FLIPs were markedly elevated in the brains of CBE-treated *RIP3*^*+/−*^mice, and these results were similar to those obtained in the *Gba*^*flox/flox*^; nestin-Cre mouse model.^87^ Researchers observed improvements in motor coordination and lifespan in CBE-treated *RIP3*^*−/−*^ mice than CBE-treated *RIP3*^*+/−*^ mice before appearance of neuronal loss but after appearance of neuroinflammation, which were accompanied by markedly fewer activated microglia in layer V of the cortex in CBE-treated *RIP3*^*+/−*^ mice.^87^ These results implicate that RIP3 pathway may participate in neuroinflammation. This study indicates that necroptosis and neuroinflammation are both involved in the pathway of pathological events in severe forms of GD, and that RIP3 is a key factor of necroptotic cell death but also participate in the pathological processes of neuroinflammation. The role of necroptosis in GD merits further investigation.

## Conclusions

Necroptosis is a novel form of programmed necrosis that can be triggered by a variety of stimuli from extracellular and intracellular. The RIP1-RIP3-MLKL necrosome plays a critical role in the initiation of necroptotic cell death. Increasing evidence suggests that inhibition of necroptosis can confer neuroprotective effect in neurodegenerative disorders, therefore indicating a promising therapeutic target. Continued investigation into the role of necroptosis in neurodegenerative diseases could provide invaluable insights into the neuron death mechanisms. Given that necroptosis may interact with other pathogenic mechanism such as apoptosis and inflammation in neurodegenerative disorders, combination therapies will be required to target the multiple activated cell death pathways to prevent the death of neurons.

To date, no suitable inhibitors of the necroptosis pathway with activity in the brains of mice or humans have been identified. Although Nec-1 crosses the blood-brain barrier, it has a short half-life of about 1 h, making it unsuitable for treatment of chronic diseases.^76^ According to the study of Takahashi *et al.*, Nec-1s might be a perfect alternative inhabitor to RIP1 and offer a promising therapeutic option for the treatment of pathological conditions involving RIP1/RIP3-dependent necroptosis.^30^ Moreover, emerging evidence showed that RIP1, RIP3 and MLKL are not the special marker of necroptosis, but also invloved in other pathways such as inflammation.^7, 92^ Researches that studied the role of necroptosis in disease using inhibitor of RIP1, RIP3, or MLKL may not precise as thought. Many conclusions drawn on the use of RIP1/RIP3/MLKL inhibitors or deficiency to explore the role of ncroptosis in neuronal diseases without premeditate the facts that they may be involved in other pathological process may require reevaluation. RIP3 inhibitors^31^ and an inhibitor of MLKL^9^ have demonstrate effect *in vitro* and *in vivo*. Development of such croptotic signaling inhibitors may pave the way for alternative therapeutic approaches for neurodegenerative diseases, for which innovative treatments are urgently required.

## Figures and Tables

**Table 1:** Inhibitors that interfere with necroptosis

**Regulatory factor**	**Synthetic inhibitor**	**References**
RIP1	Necrostatin-1(Nec-1), Nec-1s, Necrostatins	^4, 29, 30^
RIP3	GSK843, GSK872	^31^
MLKL	Necrosulfonamide (NSA)	^9, 32^

**Table 2:** Necroptosis in neurodegenerative diseases

**Diseases**	**Role of necroptosis**	**References**
Alzheimer's disease	Nec-1 improved neurobehavior in Al-treated mice model and increased survival of Al-induced neural cell death.	^47, 48^
	Nec-1 alleviated cognitive impairment with reduction of A*β* and tau abnormalities in APP/PS1 mice model.	
Parkinson disease	Nec-1 ameliorated 6-OHDA treated PC12 cells survival.	^58^
Amyotrophic lateral sclerosis	Nec-1, NSA and *RIP3* knockdown improved neuron viability in ALS astrocyte hES-MNs coculture system.	^45, 62^
	Increased expression of RIP1, RIP3 and MLKL in *OPTN^−/^^−^* mice, post-mortem patient spinal cords.	
	Mutantion of *RIP1* and *RIP3* in *OPTN^−/^^−^* mice increased neuron viability.	
Huntington disease	Nec-1 increased ST14A 8plx celll survival.	^44^
	Nec-1 improved R6/2 mice behavior and delayed symptom onset.	
Niemann-Pick disease	Nec-1 prolong cell viability in NPC1 fibroblasts and NPC1 iPS-derived neuron.	^82^
	Nec-1 delayed cerebellar Purkinje cell loss, significantly prolonged lifespan.	
	Increased levels of RIP1, RIP3 and MLKL in *NPC1^−/^^−^* mice and post-mortem patient tissues.	
Gaucher’s disease	level of RIP1, RIP3, and c-FlipS were markedly elevated in *Gba*^*flox/flox*^; nestin-Cre mice.	
	Lifespan significantly extended in *RIP3^−/^^−^* mice injected with CBE than widetype mice.	^88^
